# Regulation of lung oxidative damage by endogenous superoxide dismutase in sepsis

**DOI:** 10.1186/2197-425X-2-17

**Published:** 2014-05-23

**Authors:** Larissa Constantino, Renata Casagrande Gonçalves, Vinícius Renê Giombelli, Cristiane Damiani Tomasi, Francieli Vuolo, Luiza Wilges Kist, Giovanna Medeiros Tavares de Oliveira, Matheus Augusto de Bittencourt Pasquali, Maurício Reis Bogo, Thais Mauad, Adolfo Horn, Karen V Melo, Christiane Fernandes, José Cláudio Fonseca Moreira, Cristiane Ritter, Felipe Dal-Pizzol

**Affiliations:** Laboratório de Fisiopatologia Experimenta e Instituto Nacional de Ciência e Tecnologia Translacional em Medicina (INCT-TM), Programa de Pós-Graduação de Ciências da Saúde, Universidade do Extremo Sul Catarinense, Avenida Universitária, 1105, Criciúma, SC 88806-000 Brazil; Laboratório de Biologia Genômica e Molecular e INCT-TM, Faculdade de Biociências, Pontifícia Universidade Católica do Rio Grande do Sul, Avenida Ipiranga, 6681, Porto Alegre, RS 90619-900 Brazil; Departamento de Bioquímica, Centro de Estudos em Estresse Oxidativo, ICBS, Universidade Federal do Rio Grande do Sul, Rua Ramiro Barcelos, 2600-Anexo, Porto Alegre, RS CEP 90035-003 Brazil; Departmento de Patologia, Faculdade de Medicina, Universidade de São Paulo, São Paulo, SP 05508-070 Brazil; Laboratório de Ciências Químicas, Universidade Estadual do Norte Fluminense, Campos dos Goytacazes, RJ 28013-602 Brazil

**Keywords:** Sepsis, SOD, NO, Lung, Redox state, SOD mimetic

## Abstract

**Background:**

The purpose of this research is to study the relationship between superoxide dismutase (SOD) and lung redox state in an animal model of sepsis.

**Methods:**

Sepsis was induced in rats by the cecal ligation and perforation model (CLP). After 3, 6, and 12 h, CLP protein content and expression of SOD1, SOD2, and SOD3 were evaluated, and SOD activity was assessed*.* Oxidative damage was determined by quantifying nitrotyrosine content. Lung localization of SOD3 was performed by immunohistochemistry. The protective effect of a SOD mimetic on oxidative damage, inflammation, and lung permeability was assessed 12 and 24 h after sepsis induction.

**Results:**

Lung levels of SOD1 decreased 3 and 12 h after sepsis, but SOD2 and SOD3 increased, as well as SOD activity. These alterations were not associated with changes in *sod* gene expression. Nitrotyrosine levels increased 3 and 12 h after sepsis. The administration of a SOD mimetic decreased nitrotyrosine and proinflammatory cytokine levels and improved lung permeability.

**Conclusions:**

SOD2 and SOD3 increased after sepsis induction, but this was insufficient to protect the lung. Treatments based on SOD mimetics could have a role in lung injury associated with sepsis.

## Background

Several molecular mechanisms of inflammation and cellular damage have been implicated in the pathogenesis of sepsis including excessive reactive oxygen species (ROS) generation [1]. Main sources of ROS in the lung during sepsis are inflammatory cells and mitochondria [2, 3]. Production of ROS leads to lipid, protein, and extracellular matrix damage, which increases pulmonary inflammation [4, 5].

Antioxidants are known to counteract the deleterious effects of ROS. Superoxide dismutase (SOD) is a component of antioxidant response and catalyzes the conversion of superoxide anions to hydrogen peroxide [6]. Three SODs are found in mammals and regulate the concentration of superoxide: a cytosolic (SOD1), a mitochondrial (SOD2), and an extracellular (SOD3), which bind to both cell surfaces and extracellular matrices [7]. *sod3* gene is highly expressed in the lung, where it plays a major protective role by controlling oxidative stress and inflammation and regulating redox homeostasis of the airways [8]. Localization of SOD3 in the lung depends on its ability to bind to the extracellular matrix by a heparan sulfate domain, which can be fragmented by oxidative damage [5, 9]. Furthermore, extracellular matrix fragments stimulate inflammatory cell migration, which is of concern since matrix components are widely distributed throughout the interstitium [9]. Proteolytic cleavage of SOD3's anchorage domain alters its tissue distribution and, consequently, the oxidant/antioxidant balance [10].

In addition, bioactivity of nitric oxide (NO) partially depends on its interaction with ROS, particularly superoxide anions [11]. NO reacts with superoxide to form peroxynitrite (ONOO^−^), which induces protein oxidation and DNA damage [12]. SOD3 prevents the inactivation of NO by superoxide; therefore, an increase in *sod3* expression in blood vessels preserves endothelial function by overcoming oxidative stresses [13]. Furthermore, several evidences suggest that SOD3 may have a protective role against inflammation [10, 14].

Thus, it was hypothesized that endogenous SOD3 could have a major role in lung defenses against oxidative stress during sepsis development. The aims of this study are (1) to determine if there is a relationship between the expression and activity of SOD and the occurrence of oxidative stress and (2) to determine if the administration of a SOD mimetic is able to prevent lung damage in an animal model of sepsis.

## Methods

### Animals

Three-month-old male Wistar rats (350 to 400 g) were obtained from our breeding colony. The rats were caged in groups of five, had free access to food and water, and were maintained on a 12-h light–dark cycle (600 to 1800 hours) in a temperature-controlled colony room (22°C ± 1°C). The research protocol was approved by the Ethical Committee for Animal Experimentation of Universidade do Extremo Sul Catarinense under protocol number 21/2011.

### Cecal ligation and perforation surgery

The animals were subjected to cecal ligation and perforation (CLP) as previously described [15]. Briefly, the rats were anesthetized with ketamine (80 mg/kg). Under aseptic conditions, a 3-cm midline laparotomy was performed to allow exposure of the cecum with the adjoining intestine. The cecum was tightly ligated with a 3.0 silk suture at its base (below the ileocecal valve) and was then perforated a single time with a 14-gauge needle. The cecum was gently squeezed to extrude a small amount of feces from the perforation site into the peritoneal cavity. The animals were resuscitated with normal saline (50 mL/kg, subcutaneous) immediately and 12 h after CLP. The sham-operated group was submitted to all surgical procedures, but the cecum was neither ligated nor perforated. The animals were killed by decapitation at 3, 6, and 12 h after surgery, and the lung was removed for subsequent analyses. The number of animals in each group per experiment was ten. In some experiments, a SOD mimetic was administered once by intra-tracheal instillation immediately after CLP induction (50 mg/kg), and the lung was removed 24 h after for subsequent analyses. This mimetic has been previously described by Horn et al. [16].

### Total SOD activity

The SOD activity was measured by inhibition of adrenaline auto-oxidation followed by spectrophotometry as previously described [17].

### Immunoblotting

The lung samples were lysed in Laemmli sample buffer (62.5 mM Tris–HCl, pH 6.8, 1% (*w/v*) SDS, 10% *v/v*) glycerol). Protein (30 μg) was fractionated by SDS-polyacrylamide gel electrophoresis and then electroblotted onto nitrocellulose membranes. Protein loading and electroblotting efficiencies were verified by Ponceau S staining. The membrane was blocked in Tween-Tris-buffered saline (TTBS, 100 mM Tris–HCl, pH 7.5, containing 0.9% NaCl and 0.1% Tween-20) containing 5% albumin. The membranes were incubated overnight at 4°C with rabbit polyclonal antibody, targeting SOD1 (Santa Cruz Biotechnology, CA, USA) (dilution range 1:400), SOD2 (Santa Cruz Biotechnology) (dilution range 1:400), SOD3 (Santa Cruz Biotechnology) (dilution range 1:750), iNOS (Santa Cruz Biotechnology) (dilution range 1:400) or anti-β-actin 1:2000, in the presence of 5% milk. Thereafter, the membranes were washed with TTBS. Anti-rabbit immunoglobulin G (IgG) peroxidase-linked secondary antibody was incubated (1:10,000 dilution range), and the immunoreactivity was detected by enhanced chemiluminescence using ECL Plus kit (Thermo Fisher Scientific Inc., Pittsburgh, PA, USA). Densitometric analyses of the films were performed with ImageQuant software (GE Healthcare Life Sciences, Billerica, MA, USA). The blots were developed such that the signals are linear and non-saturating which are required for densitometry. All results were expressed as a relative ratio comparing the immunocontent of SOD1, SOD2, SOD3, and iNOS with that of the β-actin internal control [18].

### Enzyme-linked immunosorbent assay to 3-nitrotyrosine contents

An indirect enzyme-linked immunosorbent assay (ELISA) was performed to analyze the changes in 3-nitrotyrosine content. Briefly, an anti-3-nitrotyrosine polyclonal rabbit antibody (Santa Cruz Biotechnology) was diluted 2,000-fold in PBS with 5% albumin according to the manufacturer's instructions. Then, microtiter plates (96 wells, with flat bottom) were coated for 24 h with the samples that had been diluted 1:2 in PBS with 5% albumin. The plates were washed four times with wash buffer (PBS with 0.05% Tween-20), and the antibody was added to each plate for 2 h at room temperature. After washing, a second incubation with anti-rabbit antibody peroxidase conjugate (diluted 1:1,000) was performed for 1 h at room temperature. After the addition of substrates, the samples were read in a plate spectrophotometer at 450 nm. The results are expressed as changes in the percentage among the groups [19].

### Semi-quantitative reverse transcription polymerase chain reaction

All transcriptional analyses were performed in the samples in which prior Western blotting experiments revealed differences in the immunocontent. The goal was to evaluate the contribution of each gene to transcriptional changes in the immunocontent of each enzyme. Total RNA was isolated from the rat lung using TRIzol® reagent (Invitrogen, Carlsbad, CA, USA), according to the manufacturer's instructions. Using a spectrophotometer, the purity of the RNA was quantified by calculating the ratio between absorbance values at 260 and 280 nm, and its integrity was confirmed by electrophoresis using a 1.0% agarose gel. Afterward, cDNA species were synthesized using ImProm-II™ Reverse Transcription System (Promega®, Madison, WI, USA), as described by the supplier's instruction. The cDNA products (1 μL) were used as a template for each polymerase chain reaction (PCR) amplification. The PCR parameters were first optimized. Thereafter, the reactions were performed, such that product detection could be performed within the linear phase of messenger ribonucleic acid (mRNA) transcript amplification for each primer pair (Table 1). PCR for the *β-actin* gene was performed in a total volume of 20 μL using 0.1 μM of each primer, 0.2 μM dNTP, 1.6 mM MgCl_2_, and 0.2 U Taq platinum DNA polymerase (Invitrogen). For PCR amplification of *sod1*, *sod2*, and *sod3*, the reaction was performed in a total volume of 25 μL using 0.2 μM of each primer, 0.2 μM dNTP, 1.6 mM MgCl_2_, and 0.25 U Taq platinum DNA polymerase (Invitrogen). The conditions for *sod1*, *sod2*, and *sod3* PCRs were as follows: initial 1-min denaturation step at 94°C, another 1-min denaturation step at 94°C, 1-min annealing step at 60°C, 1-min extension step at 72°C for 30 cycles, and a final 10-min extension at 72°C. The conditions for the *β-actin* PCR were as follows: initial 1-min denaturation step at 94°C, another 1-min denaturation step at 94°C, 1-min annealing step at 54°C, 1-min extension step at 72°C for 35 cycles, and a final 10-min extension at 72°C. For each PCR set, a negative control was included. The PCR products were then analyzed on a 1% agarose gel containing GelRed® (Biotium, Hayward, CA, USA) and visualized with ultraviolet light. The Low DNA Mass Ladder (Invitrogen) was used as a molecular marker, and the samples were normalized against the constitutively expressed *β-actin* gene. The band intensities were measured by optical densitometry analysis, and the *enzyme*/*β-actin* mRNA ratios were established for each treatment using the freeware Image J 1.37. Each experiment was repeated at least four times using RNA isolated from independent extractions.

**Table 1:** **PCR primer design**

Enzymes	Primer sequences (5’-3’)	Anneling temperature (°C)	PCR product (bp)	GenBank accession number (mRNA)
*sod1*	F-TGCGTGCTGAAGGGCGACGGTC	60	438	BC082800
	R-AATCCCAATCACACCACAAGCCAAGC			
*sod2*	F-CCTACGTGAACAATCTGAACGTCACCGAG	60	373	BC070913.1
	R-CCCAGCAGTGGAATAAGGCCTGTGG			
*sod3*	F-GCCGAGCAGAACACCTCCAACCACG	60	377	BC061861.1
	R-CGCCGCTTCTTGCGCTCCTTTG			
*β-actin*	F-TATGCCAACACAGTGCTGCTGG	54	210	NP_742006
	R-TACTCCTGCTTCCTGATCCACAT			

### Immunohistochemistry

Lung sections (sham 0 and 12 and CLP 0 and 12, *n* = 3 in each group) were deparaffinized and hydrated. To examine their histological features, the lungs were stained with hematoxylin and eosin (H&E). After blocking of endogenous peroxidase, antigen retrieval was performed in a high-temperature Tris-citrate buffer (pH 7.2). The rabbit polyclonal anti-SOD3 (Santa Cruz Biotechnology) (diluted 1:1,600) was used as the primary antibody. The Vectastin ABC Kit (Vector Laboratories, Burlingame, CA, USA) was used as the secondary antibody, and 3,3-diaminobenzidine (DAB, Sigma, St. Louis, MO, USA) was used as the chromogen. Thereafter, the sections were counterstained with Harris hematoxylin (Merck, Darmstadt, Germany). In the negative controls, the first antibody was omitted from the procedure, and the tissues were incubated with bovine serum albumin (BSA) instead.

### Cytokine determination

Lung levels of tumor necrosis factor (TNF)-α, interleukin (IL)-1β, IL-6, and IL-10 were determined by ELISA according to the manufacturer's instructions (PrepoTech, Ribeirão Preto, SP, Brazil). All samples were assayed in duplicate.

### Lung permeability assay

Permeability changes were measured by Evan's blue dye (EBD) leakage from the blood into the airways. EBD (20 mg/kg) was administered by femoral vein injection 1 h before the end of the experiments. One hour later, the mice were bled by cardiac puncture, and the pulmonary vasculature was flushed by right ventricle puncture. The pulmonary vessels were perfused with normal saline to remove EBD from the vascular spaces. The lungs were removed *en bloc* and dried at 60°C for 24 h. EBD was extracted in formamide at 37°C for 24 h and quantitated by its fluorescence intensity. The extravasated EBD concentration in lung homogenate was calculated against a standard curve.

### Statistical analysis

Data are expressed as mean ± standard deviation. The means for the different groups were compared by *t* test or one-way or two-way ANOVA followed by Tukey test, depending on the number of experimental groups. Statistical significance was assigned to *p* < 0.05.

## Results

**Figure 1:**
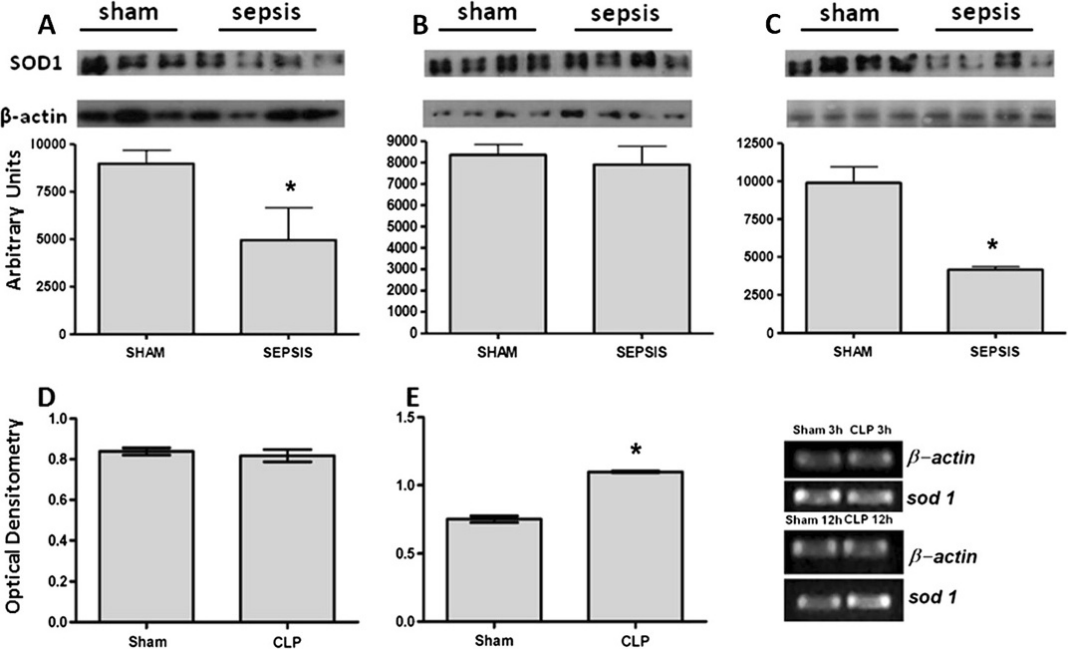
**Cytosolic superoxide dismutase (SOD1) alterations early after sepsis induction.** The animals were submitted to sepsis or sham-operated, and 3 h **(A)**, 6 h **(B)**, and 12 h **(C)** after surgery, the immunocontent of SOD1 was evaluated. The *sod1* gene expression was evaluated at 3 h **(D)** and 12 h **(E)** after surgery. The data are expressed as mean ± SD (*n* = ten animals/group). *p* value < 0.05 was considered significant when compared to the control. * denotes *p* < 0.05.

**Figure 2:**
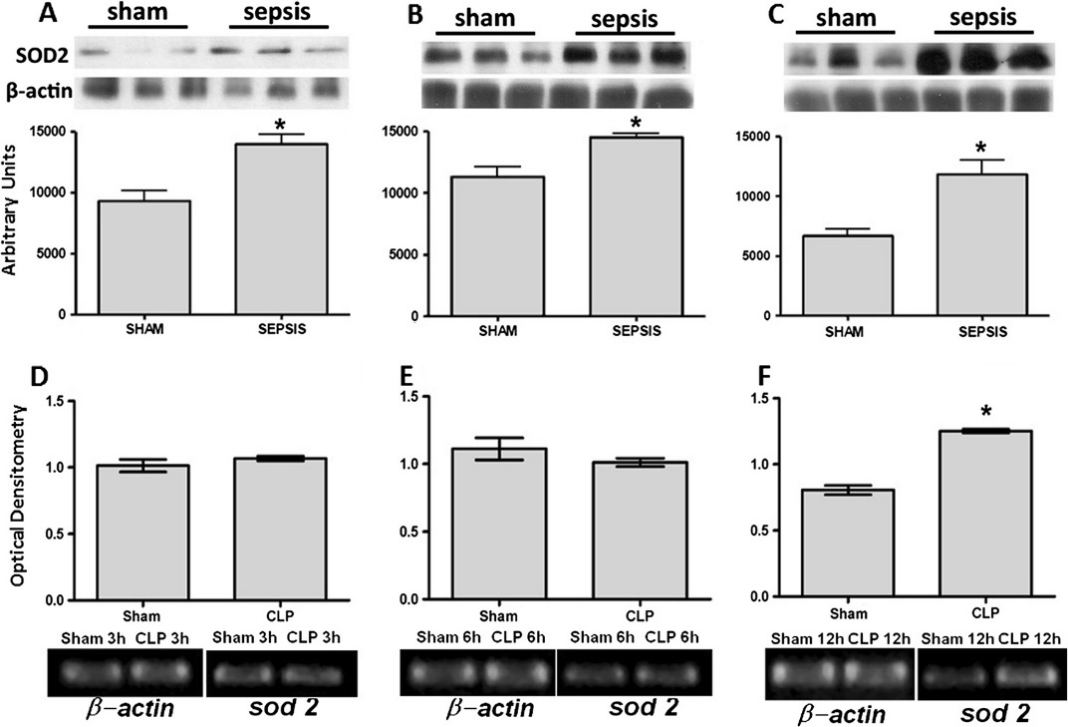
**Mitochondrial superoxide dismutase (SOD2) alterations early after sepsis induction.** The animals were submitted to sepsis or sham-operated, and 3 h **(A)**, 6 h **(B)**, and 12 h **(C)** after surgery, the immunocontent of SOD2 was evaluated. The *sod2* gene expression was evaluated at 3 h **(D)**, 6 h **(E)**, and 12 h **(F)** after surgery. The data are expressed as mean ± SD (*n* = ten animals/group). *p* value < 0.05 was considered significant when compared to the control.

**Figure 3:**
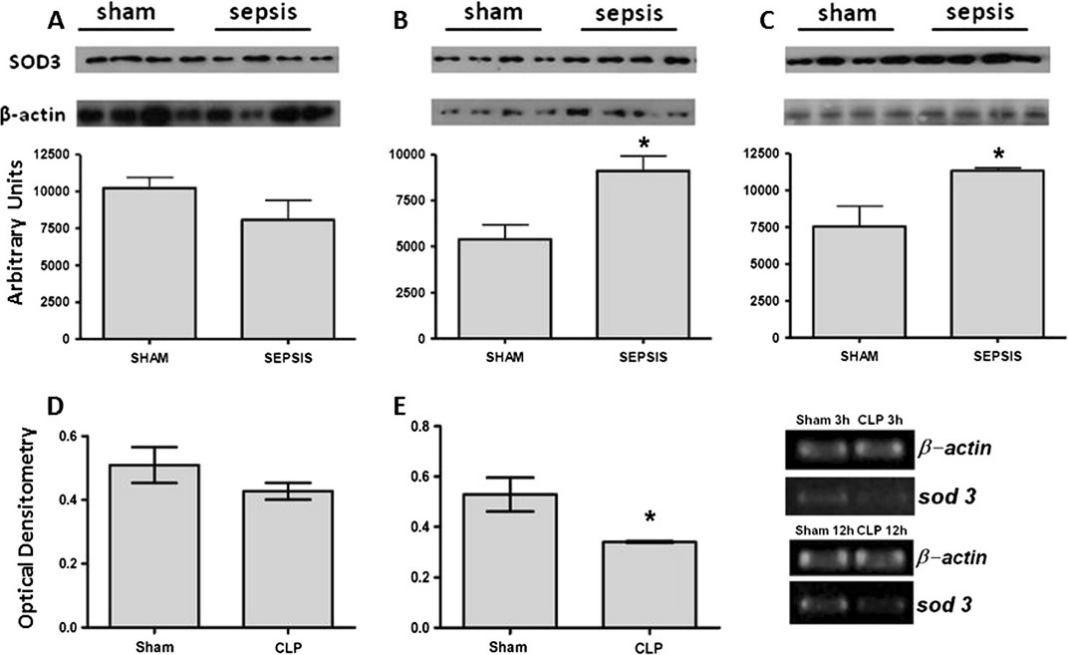
**Extracellular superoxide dismutase (SOD3) alterations early after sepsis induction.** The animals were submitted to sepsis or sham-operated, and 3 h **(A)**, 6 h **(B)**, and 12 h **(C)** after surgery, the immunocontent of SOD3 was evaluated. The *sod3* gene expression was evaluated at 3 h **(D)** and 12 h **(E)** after surgery. The data are expressed as mean ± SD (*n* = ten animals/group). *p* value < 0.05 was considered significant when compared to the control.

Levels of SOD were determined in the lung 3 to 12 h after sepsis. There was a decrease in the immunocontent of SOD1 at 3 and 12 hours, but not at 6 h, after sepsis induction (Figure 1A,B,C). Furthermore, an increase in the immunocontent of SOD2 was observed at all times (Figure 2A,B,C), and there was an increase in the immunocontent of SOD3 at 6 and 12 h after sepsis induction (Figure 3A,B,C). The SOD activity increased at 6 and 12 h (6 h, 6.03 ± 2.1 vs. 21.1 ± 5.6, *p* = 0.02; 12 h, 4.9 ± 1.7 vs. 22.3 ± 7.6 U/mg protein, *p* = 0.01) when compared to the sham and CLP animals, respectively. To determine if these changes of protein content were associated with alterations in gene expression, semi-quantitative reverse transcription polymerase chain reaction (RT-PCR) was performed. There was no consistent pattern of gene expression; while *sod1* and *sod2* gene expressions increased 12 h after sepsis (Figure 1D,E and Figure 2D,E,F), *sod3* gene expression decreased at this time point (Figure 3B).

**Figure 4:**
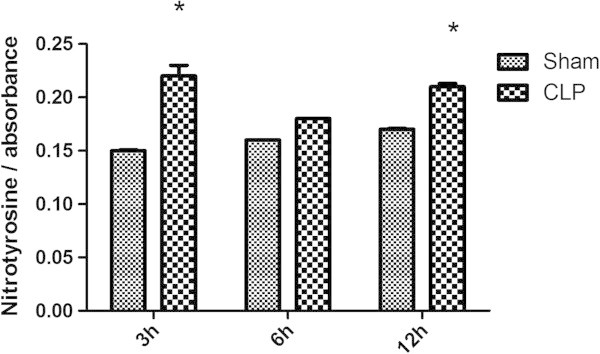
**Nitrotyrosine content early after sepsis induction.** The animals were submitted to sepsis or sham-operated, and 3, 6, and 12 h after surgery, the content of nitrotyrosine was evaluated. The data are expressed as mean ± SD (*n* = ten animals/group). *p* value < 0.05 was considered significant when compared to the control.

**Figure 5:**
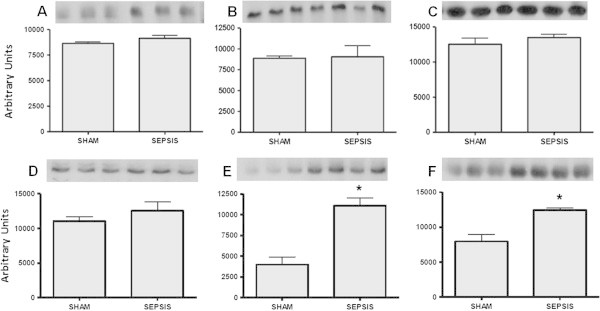
**Inducible nitric oxide synthase (iNOS) alterations early after sepsis induction.** The animals were submitted to sepsis or sham-operated, and 3 h **(A)**, 6 h **(B)**, and 12 h **(C)** after surgery, the immunocontent of iNOS was evaluated in the lung and in the pulmonary artery (3 h **(D)**, 6 h **(E)**, 12 h **(F)**). The data are expressed as mean ± SD (*n* = ten animals/group). *p* value < 0.05 was considered significant when compared to the control.

Nitrotyrosine levels increased 3 and 12 hours after CLP induction (Figure 4), suggesting that despite the increase of SOD3 content the reaction between superoxide and NO was occurring. Lung content of iNOS did not increase in the lung of septic animals (Figure 5A-C), but it was increased in the pulmonary artery (Figure 5D-E).

**Figure 6:**
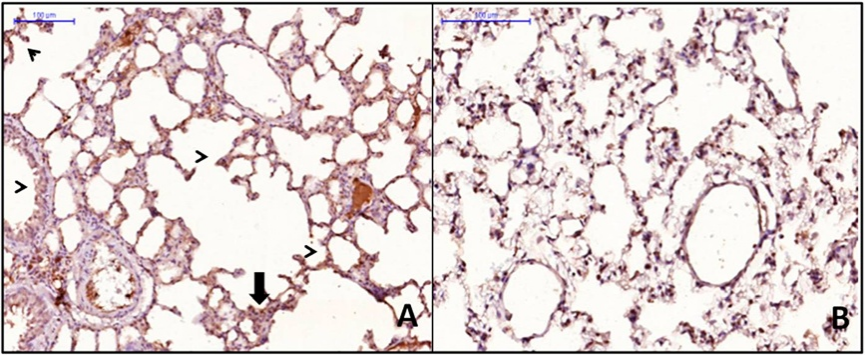
**Distribution of extracellular superoxide dismutase (SOD3) in the lung early after sepsis.** The animals were submitted to sepsis or sham-operated, and 12 h after surgery, the distribution of lung SOD3 was determined by immunohistochemistry. Representative photographs of septic **(A)** and sham **(B)** animals. Original magnification is × 6,200.

These alterations were associated with capillary congestion and infiltration of neutrophils into the alveolar septa (Figure 6). It was possible that the increase in SOD3 protein content did not protect the lung from oxidant injury due to a disorganized distribution resulting from proteolytic cleavage of its heparin-binding domain, but SOD was adequately distributed across the lung (Figure 6). It was expressed mainly in the bronchial and alveolar epithelia of the CLP animals and to a minor extent in the leukocytes and endothelial cells (Figure 6).

**Figure 7:**
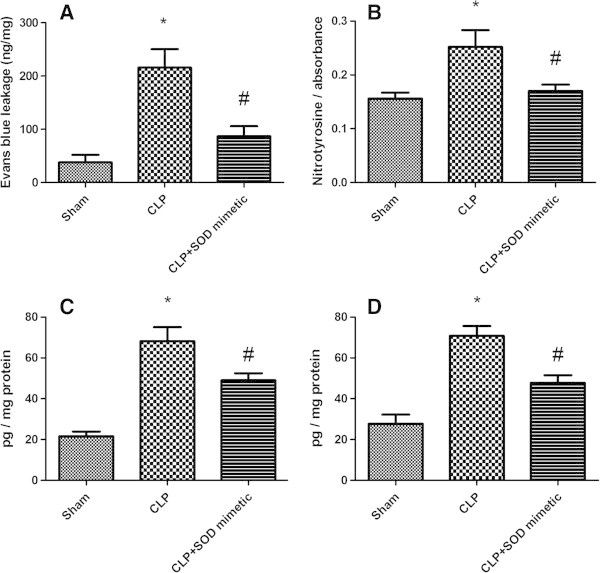
**Effects of the treatment with a SOD mimetic on lung injury induced by sepsis.** The animals were submitted to sepsis or sham-operated, and immediately after surgery, a SOD mimetic was administered once by intra-tracheal instillation. Twenty-four hours after surgery, the content of nitrotyrosine **(A)**, lung permeability **(B)**, interleukin-6 **(C)**, and tumor necrosis factor **(D)** was determined. The data are expressed as mean ± SD (*n* = ten animals/group). *p* value < 0.05 was considered significant when compared to the control.

It was expected that SOD3 protected the lung from oxidative damage and inflammation, but this was not supported by the presented data. Thus, it was administered a SOD mimetic, and it decreased the lung nitrotyrosine and cytokine levels, as well as preserved lung permeability (Figure 7A,B,C).

## Discussion

Here, in a CLP model, we demonstrated that during sepsis development, the levels of SOD2 and SOD3 increased in the lung, but these did not prevent nitrosative damage and inflammation. Furthermore, we showed that superoxide-derived lung inflammation could be attenuated by the administration of an exogenous SOD mimetic.

An increase in the levels of SOD2 and SOD3 was observed; thus, it was believed that the lung was protected from superoxide-derived oxidative damage. SOD3 is a potent inhibitor of inflammation in lung injury models [20], and mice lacking the *sod3* gene are more sensitive to lethal levels of hyperoxia [14]. Furthermore, SOD3 protects against endothelial dysfunction in mice treated with endotoxin [21]. In a murine model of emphysema, the mice that overexpress the *sod3* gene or are treated with a SOD mimetic have improved lung compliance, decreased neutrophil influx, release of proinflammatory mediators, and oxidative damage [22]. Here, we show that despite the increase in SOD content, there is an increase in lung nitrotyrosine content.

In general, inflammatory conditions (including lipopolysaccharide, hypoxia, asbestos exposure, bleomycin, and hyperoxia) induce a decrease in SOD3 content. This observation is most likely a result of proteolysis of its heparin-binding domain rather than alterations in its gene expression [20, 23]. In contrast, at earlier time points after sepsis induction, we demonstrate an increase in SOD3 levels that are properly distributed throughout the lung parenchyma, but this is not sufficient to prevent the formation of peroxynitrite. Increased oxidative damage, despite the presence of higher levels of SOD3, was described in a model of cerebral ischemia [24].

The reaction of NO with superoxide may lead to an increase in ONOO^−^, which mediates nitration of tyrosine residues in certain enzymes, including SODs. This modification leads to a reduction in their activity and, as a result, further increase in superoxide levels [25]. Specifically, ONOO^−^ is known to inactivate SOD1 and SOD2 [26, 27]. Due to the structural similarity between SOD1 and SOD3, we speculate that the activity of SOD3 may similarly be decreased by ONOO^−^. Here, we demonstrate that septic animals have increased levels of nitrotyrosine. This occurs even in the presence of higher SOD3 levels and total SOD activity. This pattern is not expected since in different models, an increase in SOD3 activity is usually associated with a decrease in ONOO^−^[28]. However, when we used an exogenous SOD mimetic, it is observed that there is a decrease in the nitrotyrosine levels. These observations suggest that the endogenous SOD system is not able to prevent oxidative damage in the CLP model. In addition, a SOD mimetic decreased the markers of lung inflammation and improved lung permeability. Moreover, these findings raise the possibility that SOD mimetics could be used to treat sepsis-related lung injury.

There are some limitations to our study. Firstly, SOD mimetic was administered immediately after CLP, when the septic response was not fully developed and animals did not presented clinical signs of severe infection. Thus, this design did not reflect the clinical scenario, but can be relevant to understand the mechanisms associated with sepsis development. Secondly, nitrotyrosine was not measured by gold-standard techniques, such as tandem mass spectrometry or high-performance liquid chromatography. Since the increase of nitrotyrosine is classically found in sepsis models, the use of semi-quantitative ELISA as a marker of oxidative damage seems to be adequate to the study aim.

## Conclusions

In conclusion, this study demonstrated an increase in SOD3 levels following sepsis; however, this was not able to prevent oxidative stress and inflammation associated with ONOO^−^ production. Nonetheless, the administration of a SOD mimetic had a positive impact, which suggests that administration of exogenous SOD may improve lung function in sepsis.
